# Preventing occupational stress in healthcare workers

**DOI:** 10.1590/1516-3180.20161341T1

**Published:** 2016-01-19

**Authors:** Jani H. Ruotsalainen, Jos H. Verbeek, Albert Mariné, Consol Serra

## Abstract

**BACKGROUND:**

Healthcare workers can suffer from occupational stress which may lead to serious mental and physical health problems.

**OBJECTIVES:**

To evaluate the effectiveness of work and person-directed interventions in preventing stress at work in healthcare workers.

**METHODS:**

**Search methods:**

We searched the Cochrane Depression Anxiety and Neurosis Group trials Specialised Register, MEDLINE, PsychInfo and Cochrane Occupational Health Field database.

**Selection criteria:**

Randomised controlled clinical trials (RCT) of interventions aimed at preventing psychological stress in healthcare workers. For work-directed interventions interrupted time series and prospective cohort were also eligible.

**Data collection and analysis:**

Two authors independently extracted data and assessed trial quality. Meta-analysis and qualitative synthesis were performed where appropriate.

**MAIN RESULTS:**

We identified 14 RCTs, three cluster-randomised trials and two crossover trials, including a total of 1,564 participants in intervention groups and 1,248 controls. Two trials were of high quality. Interventions were grouped into 1) person-directed: cognitive-behavioural, relaxation, music-making, therapeutic massage and multicomponent; and 2) work-directed: attitude change and communication, support from colleagues and participatory problem solving and decision-making, and changes in work organisation. There is limited evidence that person-directed interventions can reduce stress (standardised mean difference or SMD -0.85; 95% CI -1.21, -0.49); burnout: Emotional Exhaustion (weighted mean difference or WMD -5.82; 95% CI -11.02, -0.63) and lack of Personal Accomplishment (WMD -3.61; 95% CI -4.65, -2.58); and anxiety: state anxiety (WMD -9.42; 95% CI -16.92, -1.93) and trait anxiety (WMD -6.91; 95% CI -12.80, -1.01). One trial showed that stress remained low a month after intervention (WMD -6.10; 95% CI -8.44, -3.76). Another trial showed a reduction in Emotional Exhaustion (Mean Difference or MD -2.69; 95% CI -4.20, -1.17) and in lack of Personal Accomplishment (MD -2.41; 95% CI -3.83, -0.99) maintained up to two years when the intervention was boosted with refresher sessions. Two studies showed a reduction that was maintained up to a month in state anxiety (WMD -8.31; 95% CI -11.49, -5.13) and trait anxiety (WMD -4.09; 95% CI -7.60, -0.58). There is limited evidence that work-directed interventions can reduce stress symptoms (Mean Difference or MD -0.34; 95% CI -0.62, -0.06); Depersonalization (MD -1.14; 95% CI -2.18, -0.10), and general symptoms (MD -2.90; 95% CI -5.16, -0.64). One study showed that the difference in stress symptom level was nonsignificant at six months (MD -0.19; 95% CI -0.49, 0.11).

**AUTHORS' CONCLUSIONS:**

Limited evidence is available for the effectiveness of interventions to reduce stress levels in healthcare workers. Larger and better quality trials are needed.

The full text of this review is available free of charge from: http://onlinelibrary.wiley.com/doi/10.1002/14651858.CD002892.pub5/epdf
